# The Madness of the Millionaire

**Published:** 1892-12-17

**Authors:** 


### The Hospital

AN INSTITUTIONAL JOURNAL OF

**Science, Medicine, Nursing, and Philanthropy.**

*For Hospitals, Asylums, Infirmaries, Dispensaries, Medical Practitioners, Students, Nurses, and the Charitable Public.*

VOL. XIII.—No. 325.] [DEC. 17, 1892.

### THE MADNESS OF THE MILLIONAIRE.

THE dailies and the weeklies have all waxed eloquent about millionaires; but so far as we have seen they have universally failed to "rede the riddle aright." They have all decided that the millionaire is abnormally clever, and they have assumed that because he is clever he is sane. There could be no proof more clear and convincing that the world has not yet reached such a moderate intellectual elevation as to formulate for itself a standard of sanity which can be universally understood and accepted.

The most competent doctors have ever the highest appreciation of the value of the post-mortem table. This method of post-mortem examination is peculiar to medical men. The world has hitherto stood by a method of an entirely opposite character. *De mortuis nil nisi bonum*, it says: "Let nothing be spoken of the dead but what is for their honour." The sentiment sounds fine; and perhaps the rule it embodies ought in charity to be very generally observed. But it is not the rule of medical science; and as we are persuaded, with Mr. Gladstone, that medical science as represented by medical men is destined for leadership in the near future, it is of some importance to consider the newer rule and method which obtains on the occasion of the death of bodies committed to medical charge.

When a man dies the doctor does not say *De mortuis nil nisi bonum*, but *De mortuis nil nisi verum*; not "we will say nothing of the dead but what is good"; but, "we will say nothing of the dead but what is true." This is the complexion of modern scientific medicine. The fact is worth thinking about. Many persons will at once make the criticism that it does not matter what is said of a man's body when he is dead, whilst it may be a question of great concern what is said of his mind, his life, and his character. But such a criticism is not nearly so true as it looks. To the critical medical eye the dead man's body often reveals not only the diseased condition of the organs which were hidden during life, but the cause of their diseased condition. The post-mortem table declares that this man was a drunken debauchee, and died of alcholism; that that was the slave of illicit passion, and died of constitutional syphilis, and so on. The thing is plain as noonday to the doctor, and he records the fact as it stands. Nay, it records itself: it passes through his eye into his brain and mind; and there it stands recorded, to be obliterated no more. The physician is a recording angel whether he will or not; and the Registrar General's annual reports are strange reading to the mind which takes the just measure of them.

It is this kind of post-mortem examination to which the medical man involuntarily subjects all more than usually abnormal persons. The dead millionaire is mostly a rare phenomenon; a physiologico-moral curiosity. His wealth does not dazzle the doctor; but his astonishing power of getting it does more than dazzle. The man has aimed at money; but it is probable that he has aimed at success in his schemes still more. It is his success, his early success on a small scale; his continuous success on a larger scale; his permanent, colossal success which impresses the medical psychologist. It was a cynical remark of a wealthy, "self-made," man that "any fool can make money, but it takes a wise man to keep it." The man of many millions has made money, made always more and more money, and kept it. The central and impressive point of the phenomenon is the unvarying and unparaleled success of the man in his schemes.

Now it is worth while for even the great world to stand still for a brief period and to resolve the question of the actual status of such men in the scale of reasoning, moral animals. A millionaire is not a monstrosity; he is a type: therein lies his significance. As he is so are dozens more; and so uncounted millions fain would be. A considerable part of the world is drifting in this direction. But the true explanation of many a dead man's colossal wealth is insanity. The man was insane—insane by congenital defect; insane by voluntary and self-directed development. Most assuredly insane. Every man is insane who is absolutely destitute of moral scruples. He is below the level of the normal human intellect. He belongs to the order of highly cunning beasts. But though such a man may have been insane he was not irresponsible. He was a monomaniac: mad on one point; but with an abundance of general sanity to have kept his one mania in check. His madness consisted in the circumstance that he had no scruples. When he "went for" a thing, he went for it as the wolf goes, with absolute directness, except in so far as he was deflected by law and fear. Inner scruples to deflect him he had none; and the wolf has none. The wolf may be turned to one side by a high fence, or choked off by fear. The unscrupulous rich man may be turned aside by insurmountable difficulties, or by the terrors of the law. But his own compunctions never turn him aside. Let the consequences of his actions be what they may to other people, he carries out the actions without flinching for a single moment. Now such behaviour is madness in a man: it is not madness in a wolf; but in a man it is stark insanity.

The world makes considerable attempts to protect itself against its madmen, but its success is very limited. The real reason of this is that there is no standard of sanity which everybody accepts; and apparently very little capacity for formulating one which everybody can accept. There is a standard of honesty. "He that takes what isn't his'n, when he's catched he goes to prison," says the old saw. The stealing of a halfpenny, or of a fraction of a halfpenny, is theft, and everybody knows it to be so; and so there is no difficulty whatever in dealing with the thief. But the world is not clever enough yet to deal with insanity in so efficient a way as to protect itself. The lawyer flounders hopelessly about, and drowns his wits in a quagmire. The doctor knows a good deal more about insanity than the lawyer, and could really formulate some useful standards on the subject if he had a free hand. But the public will not give him a free hand. The "specialist" judge and the "common sense" jury are alike agreed that the doctor is to be set aside. But the doctor is right all the while. He is simply a generation or two before his time. He carries the torch of pure science in his hand; but the judges and juries lag so far behind that they do not see the light it sheds. They only hear a voice in the distance urging them forward; and if it seem to be to their immediate interest to go forward, they go; if it seem to be otherwise, they laugh derisively at the "forward" voice and remain where they are. This is bad, but it is the fate of all newly discovered truth. Time will bring about a better state of hings.

There is a moral in the millionaire's career for all sorts and conditions of men. He is practically always a failure. His schemes have succeeded; but the picture that we have of himself commonly represents him as a physical sufferer from dyspepsia and neuralgia; as a man dying in the middle of his years. He lives without happiness; he dies without honour. That is, to him at any rate, the real result of his insanity. He is ill, he is a sufferer, he does not live all his days, he dies with no more of the world's regard than a dog. He lies in his grave as poor as the poorest beggar who reads the name on his tombstone. The moral is that no man can afford to be less than a man. The unscrupulous wolf nature is for the wolf; the man it only ruins. Success is the great inspiration of worthy minds; but success with honour. Man is physiologically made for intellect, for morals, for honour, and for the success which can be achieved on these lines. Any success which is obtained by the abandonment of these is the success of the wolf, but the absolute ruin of the man.

